# Consumptive Children

**Published:** 1870-04

**Authors:** 


### CONSUMPTIVE CHILDREN.

As a general thing, too much care is taken of children who have coughs and colds. Mothers, in their anxiety to protect their children against "colds," confine them too much within doors, allowing them only to seek the air when the weather is fine, and then only after much bundling up of the body. This is particularly the case when there is any reason to suspect tubercular disease in the child. If care is taken to have the child's skin well protected from cold, by having it wear flannel drawers and undershirt, with an extra thickness of flannel about the chest; if its limbs are encased in warm, woolen stockings, thick shoes and rubber overshoes in wet weather, it is by far the best plan to allow them to run out doors *in all sorts of weather.* Consumptive children need *air*—pure air, and lots of it. This can only be had out doors, and no matter what the condition of the weather is, the air is always more wholesome than that within the living room. No harm can come to the child if it is properly clad—its lungs will always feel better, and perform their function better, in the storm, than in the illy ventilated house.

Consumptive children should have the skin sponged daily, in water of such a temperature as not to produce shock or chilliness. The body should be thoroughly dried and briskly rubbed with a coarse towel. A diet of beef steak, roast beef or mutton chops, together with baked or mashed potatoes, with milk and coarse bread, should be strictly observed. Pies, cakes and pastry must not be allowed. No grease or gravy of any sort, should be eaten. The simplest articles of food are altogether the best.

The child should sleep in a large apartment and alone. The window should be down three inches at the top and the bed so located as not to subject the sleeper to draughts of air. The underclothing should be removed at night and a thick, canton-flannel night shirt, reaching to the feet, should be worn. In the morning the sponging above referred to should be given in a moderately warm room. Then have the child don the flannels and heavy over-garments, and allow it to have a run and a romp in the open air, before breakfast.

Never send a consumptive child to school. The air in the crowded school-room will play sad havoc with its lungs. Employ a teacher at home, or, if this cannot be afforded, father and mother must act as teachers for the hour or two that the child should be confined to study, daily.

If the child is thin and puny, ask your family physician to prescribe cod-liver oil and iron, for it. A favorite formula of ours is to add two ounces of the *tincture chloride of iron* to eight ounces of pure cod-liver oil. Of this we give a child, ten years old, a teaspoonful morning and evening.

But above all, let the child live out doors as much as possible—give them warm clothing and wholesome, plain food, and we believe that very many little ones will grow to useful man and womanhood, that otherwise would find an early grave, under the kind but unwise care of a fond mother.

